# Effectiveness of Pharmacopuncture for Asthma: A Systematic Review and Meta-Analysis

**DOI:** 10.1155/2011/678176

**Published:** 2010-09-16

**Authors:** Feng-Yan Shen, Myeong Soo Lee, Sung-Ki Jung

**Affiliations:** ^1^Division of Allergy, Immune and Respiratory System, Department of Internal Medicine, College of Oriental Medicine, Kyung Hee University, Seoul 130-702, Republic of Korea; ^2^R&D Policy Team, Policy Division, Korea Institute of Oriental Medicine, Daejeon 305-811, Republic of Korea

## Abstract

Pharmacopuncture is a new needle therapy that integrates acupuncture and herbal therapies, and it has the potential to treat many diseases. A systematic review was performed to summarize and critically evaluate clinical trial evidence regarding the effectiveness of pharmacopuncture for asthma. Eight electronic databases and six journals were searched in this study. Randomized clinical trials (RCTs) in which human patients with asthma were treated with pharmacopuncture were included. The selection of studies, data extraction, and validation were performed independently by two reviewers. Four RCTs met our inclusion criteria, and the evidence from all RCTs in this study was positive. The meta-analysis showed statistically significant effects of pharmacopuncture compared to conventional treatment (*n* = 341, Risk Ratio = 1.13, 95% CI of 1.05 to 1.23, *P* = .002, heterogeneity: *χ* ^2^ = 3.55, *P* = .31, *I* ^2^ = 16%). Two trials showed favorable effects of pharmacopuncture on peak expiratory flow (PEF). However, few rigorous trials have tested the effects of pharmacopuncture on asthma. The results of our systematic review point to the potential benefits of pharmacopuncture for adults with asthma, and we suggest further RCTs and the development of a standard method of pharmacopuncture therapy.

## 1. Introduction

Asthma is a worldwide problem with an estimated 300 million affected individuals. The World Health Organization (WHO) has estimated that 15 million disability-adjusted life years are lost annually due to asthma, representing 1% of the total global disease burden. Annual worldwide deaths from asthma have been estimated at 250,000, and mortality does not appear to correlate with prevalence [1]. Asthma is a chronic inflammatory disorder of the airways. Although there are many allopathic treatments, including bronchodilators and corticosteroids, which either focus on long-term control or immediate relief, there is no single medication that is effective against both the inflammatory and bronchoconstrictive components of asthma. Therefore, many sufferers turn to alternative or complementary therapies, typically in conjunction with their conventional medications [2].

Pharmacopuncture, or herbal acupuncture (integrated acupuncture and herb therapies), is one of the new acupuncture therapies widely used in traditional East Asian medicine. An herbal extract is injected into acupuncture points to cure certain diseases [3]. Pharmacopuncture is much more effective than acupuncture alone, and its effects vary with the herbs used [4, 5]. This type of therapy is similar to acupoint injection therapy or aqua acupuncture in traditional Chinese medicine (TCM). However, acupoint injection therapy uses herbs, medicines, self-blood, oxygen, and allergens [4]. The indications for pharmacopuncture are diverse [3], and the clinical usefulness of herbal acupuncture is especially good in disorders of the musculoskeletal system [6]. Choi et al. suggested that hominis placenta extract (HPE) pharmacopuncture is effective for allergic diseases, including asthma, and autoimmune diseases. However, this study was not able to investigate the specific effect of HPE pharmacopuncture on asthma [7]. As there is no other systematic study of pharmacopuncture as an asthma treatment, the aim of this systematic review was to summarize and critically evaluate clinical trial evidence regarding the effectiveness of pharmacopuncture for asthma.

## 2. Methods

### 2.1. Data Sources

The following electronic databases were searched from inception to November 2009: PubMed, CINAHL, Medline, Cochran Library, three Korean Databases (KoreaMed, KISS, and DBPIA), and one Chinese Medical Database (CNKI). The first search term was “asthma”, and the second term was “pharmacopuncture”, “herbal acupuncture”, “acupoint”, “acupuncture point”, “aqua acupuncture”, or “aquapuncture”. The third search term was “injection” or “infusion”. We combined these three terms for the electronic search. Corresponding Korean or Chinese terms were also used. In addition, we manually searched the following Korean journals from inception to November 2009: The Korean J. Meridian and Acupoint, J. Pharmacopuncture Institute, The Korean J. Acupuncture and Moxibution Society, Korean J. Orient Int. Med, J. Korean Oriental Med, and J. Korean Orient Pediatrics. References were addressed in the original articles, and reviews were further searched for relevant studies. Dissertations and abstracts were also included.

### 2.2. Study Selection

All randomized controlled trials (RCTs) in which human patients with asthma were treated with pharmacopuncture or herbal acupoint injection were included. Studies that incorporated other diseases were excluded. Trials using other injections, such as local anesthetics, steroids, oxygen, allergens, and self-blood, were excluded. Trials in which pharmacopuncture therapies were combined with other therapies were excluded. However, trials that had the same concomitant treatments in the conventional group and the control group or had pharmacopuncture therapy in addition to the control group therapy were included. Also, studies using interventions of unproven efficacy in the control group were excluded.

### 2.3. Data Extraction and Quality Assessment

Hard copies of all articles were obtained and read in full. All articles were read by two independent reviewers (F. Y. Shen and M. S. Lee), and data from the articles were validated and extracted according to predefined criteria (Table 1). Risk of bias was assessed using the Cochrane classification for the following four criteria: sequence generation, blinding, incomplete outcome measures, and allocation concealment [8]. As it is very hard to blind therapists to the use of pharmacopuncture therapy, we assessed patient and assessor blinding separately. Disagreements were resolved by discussion between the two reviewers (F. Y. Shen and M. S. Lee). There were no disagreements between the reviewers about risk of biases. 

### 2.4. Data Synthesis

To summarize the effects of pharmacopuncture on outcomes, we used the Cochrane Collaboration's RevMan software for Windows (Version 5.0, Review Manager, RevMan; Copenhagen: The Nordic Cochrane Centre) to abstract the risk estimates (relative risk: RR). Weighted mean differences (WMDs), standard mean differences (SMDs) and the 95% confidence interval (CI) were also calculated for continuous data. For studies with insufficient information, we contacted the primary authors to acquire and verify data when possible. As suggested by the Cochrane Collaboration's software, the variance was input using a correlation factor of 0.5 [9]. If appropriate, we then pooled the data across studies using random effects models if excessive statistical heterogeneity did not exist. The chi-square test, tau^2^ test, and the Higgins *I* ^2^ test were used to assess heterogeneity.

## 3. Results

### 3.1. Study Description

**Figure 1:**
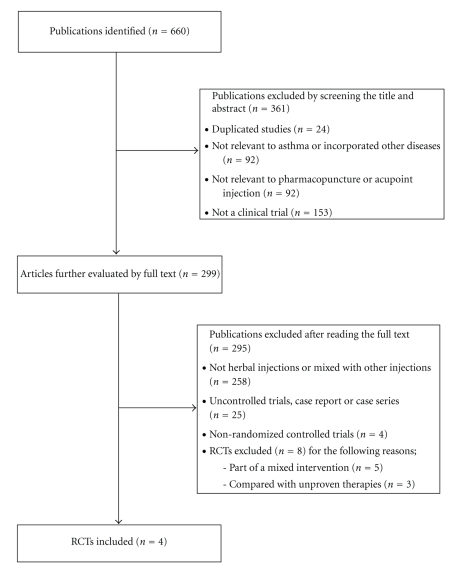
Flowchart of trial selection process. RCT: randomized clinical trial.

The literature searches revealed 660 articles, of which 656 studies were excluded (Figure 1). Four RCTs met our inclusion criteria, and their key data are listed in Table 1. All RCTs [10–13] originated from China, and all trials adopted a two-armed parallel group design. Together, they included a total of 385 patients. The patients were all adults, and the duration of disease ranged from 0.5–49 years. All levels of asthma severity were represented in the patients. One study analyzed patients during catabasis [12], and the rest [10, 11, 13] included patients with exacerbations. Duration of treatment in one trial was two years [12], and other trials [10, 11, 13] ranged from 10–15 days. There were two trials that used single-herb pharmacopuncture [10, 12]. The others used herbal formula compounds [11, 13]. *Radix Astragali *was used as the ingredient of injection in three trials [10–12]. The acupoints selected in the studies were those commonly used for asthma therapy based on TCM theory, and the following two acupoints overlapped: BL13 in three trials [10, 12, 13] and RN22 in two trials [11, 13]. One trial [11] used a single acupoint, and the other trials [10, 12, 13] used several points.

### 3.2. Study Quality

All of the included RCTs had a high risk of bias. They did not report methods of sequence generation, incomplete outcome measures, or allocation concealment.

### 3.3. Outcomes

Wangand Fu[10] tested the effect of pharmacopuncture therapy in addition to inhalation therapy. The symptom response rate in the combined therapy group was 91.4% whereas the rate was 76.3% in the inhalation-only treated group (*P* < .01). The forced vital capacities (FVCs) for the combined group and the treated group were 3.18 ± 0.88 L and 2.70 ± 0.76 L (*P* < .01), respectively. Also, the forced expiratory volumes in 1 second (FEV_1_) for the combined group and the treated group were 2.08 ± 0.69 L and 1.77 ± 0.85 L (*P* < .01), respectively. Finally, the peak expiratory flows (PEFs) for the combined group and the treated group were 3.02 ± 0.97 L/sec and 2.66 ± 0.73 L/sec (*P* < .01), respectively.

Lu and Tang [11] tested the effect of pharmacopuncture therapy in addition to conventional asthma therapies. The symptom response rate in the conventional plus pharmacopuncture therapy group was 97.1% whereas it was 76.5% in the control group (*P* < .05).

Liang et al. [12] also tested the effects of pharmacopuncture therapy in addition to conventional asthma therapies. The symptom response rate in the pharmacopuncture therapy group was 97.14% whereas the rate was 88.57% in the control group (*P* < .01). The difference between post-treatment and prior-treatment of FVC in the combination therapy group were 2.38 ± 0.52 L versus 0.21 ± 0.50 L (*P* < .01) for the control group. The difference between post-treatment and prior-treatment values of FEV_1_ in the combination therapy group were 1.67 ± 0.58 L versus 0.64 ± 0.60 L (*P* < .01) for the control group.

Tong [13] compared the effects of pharmacopuncture therapy and inhalation therapy in addition to conventional therapy. The symptom response rate in the pharmacopuncture therapy group was 95% whereas the rate was 90% in the inhalation therapy group (*P* > .05). PEFs for the pharmacopuncture and inhalation therapy groups were 360.98 ± 73.03 L/min and 346.96 ± 70.48 L/min (*P* < .05), respectively.

### 3.4. Response Rate

**Figure 2:**
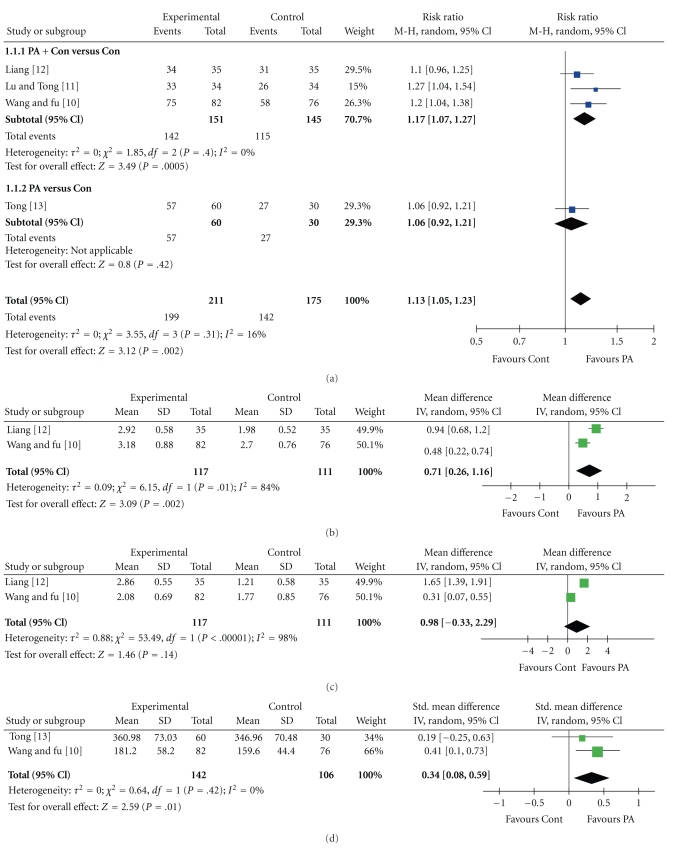
Forest plot of pharmacopuncture for asthma symptoms. (a) Response rate; (b) FVC; (c)FEV_1_; (d) PEF. FVC: forced vital capacity; FEV_1_: forced expiratory volume in 1 second; PEF: peak expiratory flow; PA: pharmacopuncture; Cont: control.

All RCTs [10–13] compared pharmacopuncture with conventional treatment. The meta-analysis showed statistically significant effects of pharmacopuncture compared with conventional treatment (*n* = 341, RR = 1.13, 95% CI of 1.05 to 1.23, *P* = .002, heterogeneity: *χ* ^2^ = 3.55, *P* = .31, *I* ^2^ = 16%, Figure 2(a)). Subgroup analyses also showed beneficial effects of conventional treatment drugs plus pharmacopuncture as compared with conventional treatments alone (*n* = 257, RR = 1.17; 95% CI of 1.07 to 1.27, *P* = .002, heterogeneity: *χ* ^2^ = 1.85, *P* = .40, *I* ^2^ = 0%, Figure 2(a)).

### 3.5. Ventilation

Two RCTs [10, 12] compared the effects of conventional treatments plus pharmacopuncture and conventional treatments alone on FVC and FEV_1_. These two RCTs reported that FVC significantly improved with conventional treatments plus pharmacopuncture. The extent of statistical heterogeneity prevented a meaningful meta-analysis of the two trials (*n* = 228, WMD = 0.71; 95% CI of 0.26 to 1.16, *P* = .002, heterogeneity: *χ* ^2^ = 6.15, *P* = .002, *I* ^2^  = 84%, Figure 2(b)). These two RCTs also reported that conventional treatments plus pharmacopuncture significantly improved FEV_1_ as compared with conventional treatments alone. However, the meta-analysis of these two RCTs failed to show favorable effects of conventional treatments plus pharmacopuncture on FEV_1_ compared with conventional treatments alone (*n* = 228, WMD = 0.98; 95% CI of −0.33 to 2.29, *P* = .14). Marked heterogeneity was observed in this model (*χ* ^2^ = 53.49, *P* < .00001, *I* ^2^ = 98%, Figure 2(c)).

Two RCTs [10, 13] tested conventional treatments plus pharmacopuncture compared with conventional treatments alone on PEF. One RCT reported beneficial effects of pharmacopuncture on PEF whereas the other RCT failed to show any benefits. However, the meta-analysis showed favorable effects of pharmacopuncture on PEF (*n* = 248, WMD = 0.34; 95% CI of 0.08 to 0.59, *P* = .01, heterogeneity: *χ* ^2^ = .64, *P* = .42, *I* ^2^ = 0%, Figure 2(d)).

## 4. Discussion

The evidence from all RCTs in this study was positive. However, few rigorous trials have tested the effects of pharmacopuncture on asthma. Furthermore, the number of trials and the total sample size are too small to draw any firm conclusions. Overall, our findings suggested the effectiveness of pharmacopuncture for asthma.

All of the RCTs had high risk of bias. Specifically, although randomization was mentioned, the details of the randomizing methods were not clear. None of the RCTs reported dropout, blinding, or allocation concealment. Although most of the RCTs had positive results, these low-quality trials are more likely to overestimate the size of the effect [14]. Such low quality trials are the main barrier to making a firm conclusion. Moreover, all of the RCTs in this study were conducted in China, a country that rarely publishes negative results [15]. These limitations may lead to the positive results in this study.

Another limitation is the design of some of these RCTs. There are three RCTs [10–12] designed as A + B versus B. Due to their design features, they were prone to false-positive results. The effects of these specific therapies are unable to be demonstrated in these RCTs [16]. The remaining RCT [13] showed significant effects of pharmacopuncture on asthma in comparison to inhalation therapy. However, both the intervention group and the control group concurrently received conventional therapy. 

All RCTs analyzed symptom response rate as a subjective outcome, but the details of symptom response rate were not unified. Importantly, FEV_1_, FVC, and PEF measurements have gained widespread acceptance for use in asthma patients over 5 years of age [17]. However, contrary to the symptom response rate, such objective measurements were reported in only a minority of trials. Another uncertain factor is that the selection of the acupoints and herbs was not consistent among trials.

According to the meta-analyses, we found significant effects of pharmacopuncture on response rate as compared with conventional treatment. The extent of statistical heterogeneity prevented a meaningful meta-analysis of the two trials [10, 12] that compared the effects of pharmacopuncture plus conventional treatments on FVC. Two trials [10, 13] showed favorable effects of pharmacopuncture on PEF. 

From our results, we cannot prove that the specific therapeutic effects of pharmacopuncture on asthma are due to their design features, but we found that pharmacopuncture can improve the effectiveness of conventional treatments as an adjunctive therapy. Furthermore, we found 300 clinical studies of acupoint injection therapy for asthma, and 91 used herbs or herb mixtures along with other injections. This finding indicates that acupoint injection therapy is widely used for asthma and is also becoming an important therapy with herbal acupoint injection or pharmacopuncture. To be classified as a complementary and alternative medicine, pharmacopuncture as well as other alternative or complementary therapies was used mostly in conjunction with allopathic medications [18, 19]. 

From the usage of pharmacopuncture in the clinic, we can deduce that this kind of therapy would have some effects on asthma, especially as an adjunctive therapy. However, we cannot make a firm conclusion. Therefore, the purpose of this paper was to guide future standardized studies so that they can provide conclusive evidence for the effectiveness of pharmacopuncture on asthma. Future studies should follow RCT methods. Second, a specific trial design has to be developed. For example, the intervention group should use pharmacopuncture therapy alone and interventions used in the other group should have proven efficacy. Third, a unified measurement should be developed. Fourth, a standard herb and acupoint selection must be developed. From our results, we discovered that the herb and acupoint most commonly used for treating asthma were *Radix Astragali* and BL13, respectively. Results of these future studies should provide useful data for developing a standard pharmacopuncture therapy for asthma.

In summary, there were many limitations precluding a firm conclusion on the effectiveness of pharmacopuncture in asthma sufferers. Despite this, we attempted to clearly sort our data to investigate the possibility of a pharmacopuncture treatment for asthma. However, we cannot be absolutely certain that our searches located all relevant RCTs. Furthermore, we also conducted meta-analyses that can increase power, improve precision, answer questions not posed by individual studies, settle controversies arising from conflicting results, and generate new hypotheses [8]. However, the use of statistics does not guarantee that the results are valid. In our case, conclusions must remain tentative. 

## 5. Conclusion

Our systematic review shows the potential benefit of pharmacopuncture for adults with asthma, both in acute exacerbation and catabasis. However, the total number of RCTs included in the analysis and the methodological quality were too low to draw any firm conclusions. More RCTs are needed, and a standard method of pharmacopuncture therapy for asthma should be developed.

## Figures and Tables

**Table 1:** Summary of randomized clinical trials of pharmacopuncture for asthma.

First author (year)	Wang (2008) [10]	Lu (2005) [11]	Liang (2004) [12]	Tong (2007) [13]
Stage, Levels of severity	Acute, n.r.	Acute, n.r.	Catabasis, all levels	Acute, mild persistent and moderate persistent

Patient age/duration of disease	PA: 32 ± 18.6 y/12 ± 9.8 y	A: 49 y/3.6 y	PA: 36.87 ± 8.85 y/10.11± 4.58 y	PA: 45.74 y/3.8 y
	Con: 35 ± 20.3 y/12 ± 8.5 y	Con: 47 y/2.3 y	Con: 37.58 ± 6.21 y/11.01 ± 3.34 y	Con: 41.9 y/3.63 y

Intervention (Regimen)	(A) PA (1 mL/point, q.d. for 2 weeks, *n* = 82), plus (B)	(A) PA (1 mL/point, q.d. for 10–15 days, *n* = 34), plus (B)	(A) PA (1 mL/point, 2 times/week, 3 months/session, 3 month break between 2 sessions, 4 sessions in total, *n* = 34) plus (B)	(A) PA (1.5–2 mL/point, q.d. for 10 days, *n* = 60) plus conventional therapies (anti-inflammation, spasmolysis, dyspnea relief and sputum elimination)

Control (Regimen)	(B) Bricalin inhalation (0.25–0.5 mL, t.i.d. for 2 weeks, *n* = 76)	(B) Glucocorticoid, aminophylline, acidity modulation, oxygen uptake, etc. (*n* = 34)	(B) Beclomethasone dipropionate + ventolin inhalation (q.d. for 2 years, dosage modulated depending on symptoms, *n* = 35)	(B) Bricalin inhalation (0.5 ml t.i.d. for 10 days, *n* = 30), plus conventional therapies (anti-inflammation, spasmolysis, dyspnea relief, and sputum elimination)

Study design	A + B versus B	A + B versus B	A + B versus B	A + C versus B + C

Main outcome measure	(1) PFT (FVC, FEV_1_, PEF)	Symptom response rate (degree of the asthmatic attack)	(1) Symptom response rate (degree of asthmatic attack, change in PEF% or FEV_1_%, steroids and bronchodilators required or not)	(1) Symptom response rate (blood routine examination, frequency of asthma attack, breathing rate, pulse rate, and wheezing sound)
	(2) Symptom response rate (degree of asthmatic attack, change in PEF% or FEV_1_%, steroids and bronchodilators required or not)		(2) Difference between post-treatment and prior-treatment values of FVC and FEV_1_	(2) PEF

Intergroup difference	(1) FVC, *P* < .01; FEV_1_, *P* < .01; PEF, *P* < .01	*P* < .05	(1) *P* < .01	(1) NS
	(2) *P* < .01		(2) FVC, *P* < .01; FEV_1_, *P* < .01	(2) *P* < .05

Ingredients of the injection	*Radix Astragali*	*Bufo, Radix Astragali, Semen Ginkgo, Semen Armeniacae Amarum, Radix et Rhizoma Asteris, Radix Peucedani, Fructus Schisandrae Chinensis, Radix Aconiti lateralis Praeparata, Fructus Piperis, and so forth.*	*Radix Astragali*	*Radix Angelicae Sinensis, Rhizoma Chuanxiong, Flos Carthami*

Acupoint	ST36, BL13, BL23 (one side at a time)	RN22	BL13 (one side at a time), DU14	EX-B1, BL13, RN22, SJ6, ST40 (one side at a time)

Con: control; d: day(s); m: month(s); FEV_1_: forced expiratory volume in 1 second; FVC: forced vital capacity; n.r.: not reported; NS: not significant; PA: pharmacopuncture; w: week(s); y: year(s); PEF: peak expiratory flow; PFT: pulmonary function test.
